# Universal Regimes in Long-Time Asymptotic of Multilevel Quantum System Under Time-Dependent Perturbation

**DOI:** 10.3390/e23010099

**Published:** 2021-01-12

**Authors:** Vladimir Akulin

**Affiliations:** 1 Tikhonov Institute of Mathematics and Electronics, High School of Economics, 34 Tallinskaya Ulitsa, 123458 Moscow, Russia; akulinvladimir@gmail.com; 2 Kharkevich Institute of the Russian Academy of Sciences, 19 Bol’shoi Karetnyi per., 127994 Moscow, Russia

**Keywords:** multilevel quantum system, resonant excitation, random walk, dynamic localization

## Abstract

In the framework of an exactly soluble model, one considers a typical problem of the interaction between radiation and matter: the dynamics of population in a multilevel quantum system subject to a time dependent perturbation. The algebraic structure of the model is taken richly enough, such that there exists a strong argument in favor of the fact that the behavior of the system in the asymptotic of long time has a universal character, which is system-independent and governed by the functional property of the time dependence exclusively. Functional properties of the excitation time dependence, resulting in the regimes of resonant excitation, random walks, and dynamic localization, are identified. Moreover, an intermediate regime between the random walks and the localization is identified for the polyharmonic excitation at frequencies given by the Liouville numbers.

## 1. Time Dependent Perturbation of a Quantum System

Action of a time dependent perturbation $\hat{V}\left(t\right)$ on a quantum system defined by a Hamiltonian ${\hat{H}}_{0}$ with a sufficiently rich spectrum can be considered to be a typical problem in the domain of the coherent interaction of radiation with matter. Every expert in the field can immediately give many particular examples of such a problem that have exact analytical solutions, including the most studied case of the weak perturbation regime where one calculates composite matrix elements of the multiphoton transitions and Fourier transforms of powers of the corresponding time dependencies. One also knows that for the strong perturbation regime, the general solution cannot be explicitly given as an exact analytical expression other than the well-known symbolic form

(1) $$\hat{U}\left(t\right)=T\exp\left\{-i\int \left({\hat{H}}_{0}+\hat{V}\left(t\right)\right)dt\right\}$$

of the time-ordered exponent of the integral of Hamiltonian ${\hat{H}}_{0}+\hat{V}\left(t\right)$ for the evolution operator $\hat{U}\left(t\right)$. Here one addresses the question: is it still possible to analytically find the *asymptotic* behavior of a rather complex quantum system under a strong time-dependent perturbation and qualitatively identify different regimes characterized by certain *functional properties* of this time dependence? By the strong perturbation one means here the case where a typical matrix element of the perturbation $\hat{V}\left(t\right)$ exceeds considerably a typical spacing among the successive eigen energies of the unperturbed Hamiltonian ${\hat{H}}_{0}$.

The problem has already been tackled [1,2] by the methods of the Random Matrix Theory for the case $\hat{V}\left(t\right)=f\left(t\right)$ $\hat{V}$, where both matrices $\hat{V}$ and ${\hat{H}}_{0}$ were assumed to belong to one of Gaussian Statistical Ensembles. One of the regimes identified is so-called Dynamic Localization [3], where despite the presence of all-order resonances, the quantum system does not get excited beyond a certain limit due to the complicated interference phenomena. Still, general relations among the functional properties of f(t) and the asymptotic behavior of the population distribution over the energy scale have not yet been revealed. In contrast to the statistical approach developed in these publications, the purpose of the present paper is to establish a certain type of such relations between the Fourier composition of f(t) and the asymptotic width of the population distribution over the energy scale for one particular choice of the operators $\hat{V}$ and ${\hat{H}}_{0}$. One can conjecture that this choice offers description of a rather generic situation, which is typical of the asymptotic behavior of almost all particular realizations of these operators. Discussion about the applicability of the different particular cases revealed to the generic situation is given at the last section of the present text. The result shows that in some regimes, the number theory related to the best rational approximation problem may play an important role, as it was already encountered earlier for two other models Refs. [4,5]. Note that more technical details can be found in Ref. [6].

## 2. What May Affect the Asymptotic Behavior in the Strong Perturbation Regime?

Speaking about dynamics of a quantum system with the Hamiltonian

(2) $$\hat{H}\left(t\right)={\hat{H}}_{0}+f\left(t\right)\hat{V},$$

one can identify two ingredients of the problem potentially capable of influencing the asymptotic behavior: (i) The algebraic properties of the pair of operators ${\hat{H}}_{0}$ an d $\hat{V}$, and (ii) The functional properties of the time dependence f(t). An amalgam of these two distinct types of properties can be observed in a natural way when one employs the Magnus expression

(3) $$\begin{array}{c} i\hat{S}\left(t\right)\equiv \log\left[T\exp\left\{i\int \hat{H}\left(t\right)dt\right\}\right]=\int_{0}^{t}dt_{1}\frac{\hat{H}\left(t_{1}\right)}{-i}+\frac{1}{2}\int_{0}^{t}dt_{1}\int_{0}^{t_{1}}dt_{2}\frac{\left[\hat{H}\left(t_{1}\right),\hat{H}\left(t_{2}\right)\right]}{{\left(-i\right)}^{2}}\\ +\frac{1}{6}\int_{0}^{t}dt_{1}\int_{0}^{t_{1}}dt_{2}\int_{0}^{t_{2}}dt_{3}\frac{\left[\hat{H}\left(t_{1}\right),\left[\hat{H}\left(t_{2}\right),\hat{H}\left(t_{3}\right)\right]\right]+\left[\hat{H}\left(t_{3}\right),\left[\hat{H}\left(t_{2}\right),\hat{H}\left(t_{1}\right)\right]\right]}{{\left(-i\right)}^{3}}+\ldots \end{array}$$

for the action matrix $\hat{S}\left(t\right)$ of the evolution operator Equation (1) corresponding to the Hamiltonian Equation (2), which yields

(4) $$\begin{array}{cc} \hat{U}\left(t\right) & =\exp\left\{-i{\hat{H}}_{0}t-i\hat{V}\int f\left(t\right)dt+\frac{1}{2}\left[{\hat{H}}_{0},\hat{V}\right]\left(\int \int^{t}f\left(\tau \right)d\tau dt+\int f\left(t\right)\int^{t}d\tau dt\right)+\ldots \right. \end{array}$$

(5) $$\begin{array}{cc} \; & \left.+\frac{i^{l}p}{q}\left[\left[\ldots \left[{\hat{H}}_{0},\hat{V}\right],\ldots \right],{\hat{H}}_{0}\right]\left(\int f\left(t\right)\int \ldots \int \int^{t}f\left(\tau \right)d\tau \ldots dt+\ldots \right)+\ldots \right\}. \end{array}$$

General term Equation (5) of the Magnus series for the action matrix in the exponent for $\hat{U}\left(t\right)=e^{i\hat{S}\left(t\right)}$ Equation (4) is a product of three factors: (i) an operator part $\left[\left[\ldots \left[{\hat{H}}_{0},\hat{V}\right],\ldots \right],{\hat{H}}_{0}\right]$, which is given by one of the all-order commutators of the operators ${\hat{H}}_{0}$ and $\hat{V}$ , and this part is representing the algebraic properties, (ii) a functional part $\left(\int f\left(t\right)\int \ldots \int \int^{t}f\left(\tau \right)d\tau \ldots dt+\ldots \right)$, which is given by a relevant combination of integrals constructed from the function f(t) representing the functional properties of the perturbation time dependence, and (iii) a numerical coefficient $\frac{i^{l}p}{q}$, which cannot be given as a general algebraic expression, but can be recursively calculated up to any given order.

For a system with a finite dimension *N* of the Hilbert space, successive all-order commutation of a pair of operators produces a complete set of $N^{2}-1$ linearly independent operators forming a closed algebra $A_{N}$ of a finite number $N^{2}-1$ of generators, such that no new linearly independent operators can be produced starting from a certain order of commutation. In contrast, the functional part gets more and more involved with the increasing order of commutation. The longer the elapsed time is, the higher are the orders of nonlinearity of the functional parts that give the leading contribution to the action series, and these orders grow more or less linearly with the time. In such a situation, after a certain interval of time, a linear combinations of all $N^{2}-1$ generators of the algebra $A_{N}$ will appear in the exponent Equation (4) with the coefficients given by complicated functionals of the time dependence f(t).

There exists another way to preliminary describe the same situation. Consider the problem in the interaction representation, where the perturbation matrix elements

$${\left({\hat{V}}_{\mathrm{int}}\left(t\right)\right)}_{n,m}=V_{n,m}e^{-i(E_{n}-E_{m})t}$$

acquire the phase factors suggested by the eigen energies $E_{k}$ of the unperturbed Hamiltonian ${\hat{H}}_{0}$. The operator $f\left(t\right){\hat{V}}_{\mathrm{int}}\left(t\right)$ can be directly substituted to the Magnus series Equation (3) for the evolution operator, where each element of the action matrix

$$S_{n,m}\left(t\right)=s_{n,m}F\left\{f\left(t\right),E_{n},E_{m}\right\}$$

can be seen as a factor $s_{n,m}$, which accounts for a sort of selection rules for the composite matrix elements dictated by the algebraic properties of the operators, and the functional part $F\left\{f\left(t\right),E_{n},E_{m}\right\}$, which accounts to the capability of the function f(t) to produce all-order combinational resonances between the states with the energies $E_{n}$ and $E_{m}$. It is, therefore, natural to conjecture that no matter what the pair of the operators ${\hat{H}}_{0}$ and $\hat{V}$ was originally, for strong coupling, only the type of the algebra $A_{N}$ and the functional properties of f(t) are the relevant characteristics that governs the dynamics of the system in the asymptotic regime, whereas the selection rules play no crucial role since all-order resonances are capable of coupling each pair of the state at appropriate time domains.

## 3. Choice of the Model

In order to analyze the asymptotic dynamics of a quantum system under the action of a time-dependent interaction within the framework of the aforesaid conjecture, one can make a particular choice of the operators ${\hat{H}}_{0}$ and $\hat{V}$ that allows one to find an exact analytical solution of the problem, but in the same time generates complete algebra $A_{N}$ of a given large number *N* of the elements. To gain an idea about universality of such dynamics, one has to simply consider the time intervals long enough, such that the main contribution to the Magnus series comes from the high-order commutations and therefore manifests universality of the asymptotic behavior since in this regime the particular choice no longer has important effect on the population dynamics of the system.

One can make the following choice of the operators specified by the matrix elements:

(6) $$\begin{array}{cc} {\left({\hat{H}}_{0}\right)}_{n,m} & =n\delta_{n,m}\\ {\left(\hat{V}\right)}_{n,m} & =1, \end{array}$$

which corresponds to the equidistant spectrum of the unperturbed system, and a rank 1 perturbation with all identical matrix elements equal to unity. Direct inspection shows that for a given size of the Hilbert space *N* all the rest of the set of $N^{2}-1$ linearly independent N×N matrices different from unity can indeed be obtained by high-order commutations of the pair of these operators.

The corresponding Schrödinger equation

(7) $$i{\dot{\psi }}_{n}=n\psi_{n}+f\left(t\right)\sum_{j}^{N}\psi_{j}$$

with the initial condition $\psi_{n=0}\left(t=0\right)=1$ can be re-written in the form

(8) $$\psi_{n}\left(t\right)=\delta_{n,0}-i\int_{0}^{t}d\tau e^{-in(t-\tau )}f\left(\tau \right)\sum_{j}^{N}\psi_{j}\left(\tau \right),$$

which for the combination $Q\left(t\right)=\sum_{j}^{N}\psi_{j}\left(t\right)$ results in an integral equation

(9) $$Q\left(t\right)=1-i\int_{0}^{t}d\tau G\left(t-\tau \right)f\left(\tau \right)Q\left(\tau \right).$$

In the limit of large *N*, the kernel

(10) $$G\left(z\right)=\sum_{n}^{N}e^{-inz}$$

of this equation acquires the form

(11) $$G\left(z\right)=2\pi \sum_{n}\delta \left(z-2\pi n\right)$$

of an infinite sequence of 2π-periodic δ-shaped spikes, while for a finite *N* these spikes have a finite width. One may say that this kernel accounts for the 2π-periodic returns of the population amplitudes back to the interacting state given by the linear combination $\sum_{j}^{N}\psi_{j}\left(\tau \right)$ as the result of action of the unperturbed Hamiltonian Equation (6). The combination

(12) R(t)=f(t)Q(t)

governs the probability amplitude distribution at a given time via the Fourier transform of $\Theta \left(t-\tau \right)R\left(\tau \right)$, as it follows from Equation (8).

## 4. The Solution and the Required Functional Characteristics of the Time Dependence

Equation (9) with the kernel Equation (11) is actually an algebraic relation among the values of the function Q(t) with the times arguments shifted each with respect to another by multiples of the period 2π, which is

(13) $$Q\left(t\right)=1-i\pi f\left(t\right)Q\left(t\right)-2\pi i\sum_{k=1}^{\mathrm{ip}\left\{t/2\pi \right\}}f\left(t-2\pi k\right)Q\left(t-2\pi k\right),$$

where $\mathrm{ip}\left\{t/2\pi \right\}$ stands for the integer part of the ratio t/2π. Let us denote by $Q_{n}\left(\theta \in \left\{0,2\pi \right\}\right)$, $R_{n}\left(\theta \in \left\{0,2\pi \right\}\right)$ and $f_{n}\left(\theta \in \left\{0,2\pi \right\}\right)$ the value of the function Q(t), R(t) and f(t) at t=2πn+θ, respectively, and arrive at the algebraic equation

(14) $$Q_{n}\left(\theta \right)=1-i\pi f_{n}\left(\theta \right)Q_{n}\left(\theta \right)-2\pi i\sum_{k=1}^{n-1}f_{k}\left(\theta \right)Q_{k}\left(\theta \right),$$

which has an explicit solution

(15) $$Q_{n}\left(\theta \right)=\frac{1}{1-i\pi f_{n}\left(\theta \right)}\prod_{k=0}^{n}\frac{1-i\pi f_{k}\left(\theta \right)}{1+i\pi f_{k}\left(\theta \right)}.$$

This expression and Equation (8) yields explicit expression for the probability amplitudes at the time points t=2πr

(16) $$\begin{array}{cc} \psi_{m}\left(2\pi r\right) & =-i\int_{0}^{2\pi }d\theta e^{-im\theta }\sum_{n=0}^{r}f_{n}\left(\theta \right)Q_{n}\left(\theta \right)=i\int_{0}^{2\pi }d\theta e^{-im\theta }\sum_{n=0}^{r-1}\frac{-f_{n}\left(\theta \right)}{1-i\pi f_{n}\left(\theta \right)}\prod_{k=0}^{n}\frac{1-i\pi f_{k}\left(\theta \right)}{1+i\pi f_{k}\left(\theta \right)} \end{array}$$

(17) $$\begin{array}{cc} \; & =-\int_{0}^{2\pi }\frac{d\theta }{2\pi }e^{-im\theta }\sum_{n=0}^{r-1}\left(\prod_{k=0}^{n}\frac{1-i\pi f_{k}\left(\theta \right)}{1+i\pi f_{k}\left(\theta \right)}-\prod_{k=0}^{n-1}\frac{1-i\pi f_{k}\left(\theta \right)}{1+i\pi f_{k}\left(\theta \right)}\right)=\int_{0}^{2\pi }\frac{d\theta }{2\pi }e^{-im\theta }\left(1-\prod_{k=0}^{r-1}\frac{1-i\pi f_{k}\left(\theta \right)}{1+i\pi f_{k}\left(\theta \right)}\right). \end{array}$$

When speaking about population of the states that have energies far enough from the energy of the initially populated state, the index *m* is assumed to be large. That is why the last term 1 in the integrand of Equation (17) is going to be ignored hereafter. For a function f(t) with a restricted spectral width, the principle contribution in the integrand of (17) is than given by the product

(18) $$\prod_{k=0}^{r-1}\frac{1-i\pi f_{k}\left(\theta \right)}{1+i\pi f_{k}\left(\theta \right)}$$

with a large *r*. In fact, in order to ensure a substantial population of such states, the expression in parenthesis as a function of θ should change rapidly and therefore contain rapidly oscillating harmonics in their Fourier decomposition, which can only be the case when the product Equation (18) of the phase factors

(19) $$\frac{1-i\pi f_{k}\left(\theta \right)}{1+i\pi f_{k}\left(\theta \right)}$$

is long enough.

Please note that in the strong coupling regime, the main contribution of each of factors Equation (19) to the overall phase change of Equation (18) comes from the domains where the corresponding function $f_{k}\left(\theta \right)=f\left(\theta +2\pi k\right)$ crosses zero, and where generally speaking, the phase change is the most rapid, while outside of these domains, namely where $f_{k}\left(\theta \right)\gg 1/\pi$, the phase remains close π. When in a vicinity of a point θ two functions, say $f_{k}\left(\theta \right)$ and $f_{k^{\prime }}\left(\theta \right)$, turn to zero having same signs of the derivatives, such a coincidence yields two factors in the product of Equation (18) that rapidly change their phases in the same direction, which, evidently, increases the overall rate of the phase change in this very moment of time θ. If the signs of the derivatives are opposite, the phase change decelerates. One has thus identified a qualitative functional characteristic of f(t), which is responsible for broadening of the population distribution over the energy scale—this is the *coincidence statistics of zeros* of the function f(t) folded with the period 2π of the kernel G(t), which should be taken *with the allowance for the derivatives* $f^{\prime }\left(t\right)$ in these points.

The formal reason for this conclusion relies on the saddle-point approximation of the main contribution to the integral Equation (17)

(20) $$\psi_{m}\left(2\pi r\right)=-\int_{0}^{2\pi }\frac{d\theta }{2\pi }e^{-im\theta +\sum_{k=0}^{r-1}\log\frac{1-i\pi f_{k}\left(\theta \right)}{1+i\pi f_{k}\left(\theta \right)}},$$

for which the saddle-point equation

$$-m-\sum_{k=0}^{r-1}\frac{2\pi f_{k}^{\prime }\left(\theta \right)}{1+{\left[\pi f_{k}\left(\theta \right)\right]}^{2}}=0$$

allows one to conclude that the points where $f_{k}\left(\theta \right)=0$ indeed give maximum contributions weighted by the derivatives $f_{k}^{\prime }\left(\theta \right)$ at these points. Strictly speaking, there exists a regime where the function f(t) always remains much smaller than unity and the main role therefore play the points with the maximum derivative f(t). However, this is the regime of weak coupling, not considered here, which is adequately tractable by the regular time dependent perturbation theory.

## 5. Four Particular Regimes of the Excitation

Four particular cases can immediately be suggested for the analysis: the case (A) of a periodic f(t), with a period $2\pi \frac{p}{q}$ which is a rational fraction of the period of G(t), the case (B) of a function f(t) randomly changing for every next return period, the case (C) of an irrational ratio of the periods of f(t) and G(t), and the case (D) of several incommensurable irrational harmonics of f(t).

### 5.1. Integer and Rational Frequency Spectrum of f(t)

The simplest case corresponds to the situation where the spectrum of f(t) has integer frequencies. Each product Equation (18) is therefore simply the *r*-th power of the factor Equation (19) with k=0, and for the long time asymptotic r→∞ one can separately consider contribution of each root $f(\theta_{n})=0$, by performing Taylor expansion $f(\theta_{n})≃$ $f_{n}\left(\theta -\theta_{n}\right)$, thus obtaining Equation (20) in the form

$$\psi_{m}\left(2\pi r\right)≃\sum_{\mathrm{roots}\;n}\frac{e^{-im\theta_{n}}}{2\pi }\int_{-\infty }^{\infty }dxe^{-imx+r\log\frac{1-i\pi f_{n}x}{1+i\pi f_{n}x}},$$

where $x=\left(\theta -\theta_{n}\right)$. Evaluation by the stationary phase method yields

(21) $$\psi_{m}\left(2\pi r\right)≃\sum_{\mathrm{roots}\;n}\frac{\exp\left(ir\Phi_{n}\right)}{i\sqrt{2\pi r}z^{3/4}{\left(2\pi f_{n}+z\right)}^{1/4}},$$

where $z=\frac{m}{r}$ and $\Phi_{n}$ is a phase factor, which for positive $f_{n}$ implies negative *z* and reads

$$\Phi_{n}=\frac{\sqrt{z}\sqrt{2\pi f_{n}+z}}{i\pi f_{n}}-z\theta_{n}+\arg\frac{\sqrt{z}-\sqrt{2\pi f_{n}+z}}{\sqrt{z}+\sqrt{2\pi f_{n}+z}}.$$

For the negative $f_{n}$ and positive *z* the phase factor is similar.

From the structure of the amplitude Equation (21), where the time dependence enters via the parameter *z* exclusively, it becomes clear that in the long time limit, the population distribution along the energy scale gets broader and the distribution width scales linearly with the increasing number *r* of periods given by the integer part of the dimensional time t/2π. Zeros of $f\left(t\right)$ with the positive derivatives account for the distribution propagation towards negative energies with the velocities of $f_{n}$, while the negative ones account for the energy increase. One can interpret the result as an excitation of the equidistant spectrum of the band by a resonant field and its harmonics that are just parametrized by the positions of zeros of $f\left(t\right)$ and by the slopes of the function in these points. The resulting population distribution over the energy scale linearly broadens with time, and the velocity of this broadening are given by the typical slope $\bar{\left|f_{n}\right|}$.

When the field frequency spectrum is given not by integers, but by multiples of a rational number $\frac{p}{q}$, there exists a common period 2πq of G(t) and $f\left(t\right)$, such that one still can explicitly perform summation and write an expression for the amplitudes

(22) $${\left.\psi_{m}\left(2\pi r\right)\right|}_{r=vq+1}=\int_{0}^{2\pi }\frac{d\theta }{2\pi }e^{-im\theta }{\left(\prod_{k=0}^{q-1}\frac{1-i\pi f(\theta +2\pi k)}{1+i\pi f(\theta +2\pi k)}\right)}^{pv}$$

equivalent to Equation (20). Again, for the long time limit and the large *m*, the main contribution comes from the most rapidly oscillating first term in the parentheses representing now the net contributions during the common period 2πq, while the summation over *q* periods of the kernel G(t) results in the phase $\sum_{k=0}^{q-1}pv\log\frac{1-i\pi f(\theta +2\pi k)}{1+i\pi f(\theta +2\pi k)}$ . The latter linearly increases with the number $r∼\frac{pv}{q}$ of the perturbation periods. One finds a situation similar to the case of integer frequencies given by Equation (21), the energy distribution width increases linearly in time. This case corresponds to a parametric resonance between the equidistant spectrum frequency and the frequency of the field.

### 5.2. A Random Function f(t)

The situation is quite different if $f\left(\theta \right)\;\neq \;f\left(\theta +2\pi k\right)$ for all *k*. This is the case if the function $f\left(t\right)$ is not a periodic but a random one, which means that in the time interval (0,2π) it varies in a way different from that in the interval (2π,4π), and in a yet different way in each next time interval $\left(2\pi k,2\pi \left(k+1\right)\right)$. In other words, time dependences of the perturbation during different revival periods are completely de-correlated and their actions may compensate each other on average. Equation (17) written in the form

(23) $$\psi_{m}\left(2\pi n\right)=-\int_{0}^{2\pi }\frac{d\theta }{2\pi }e^{-im\theta +i\Phi_{n}\left(\theta \right)}$$

shows that the phase factors

(24) $$i\Phi_{n}\left(\theta \right)=\sum_{k=0}^{n-1}\log\frac{1-i\pi f(\theta +2\pi k)}{1+i\pi f(\theta +2\pi k)}$$

are no longer linearly increasing functions of the number of the periods, since the sum of *n* random functions is not *n* times but just $\sqrt{n}$ times larger that a typical term of this sum. In other words, for a given θ, not averages but only statistical fluctuations of the phases $\log\frac{1-i\pi f(\theta +2\pi k)}{1+i\pi f(\theta +2\pi k)}$ allow for the nonvanishing result, and hence the width of the population distribution over the energy scale grows according the diffusion law—as a square root of time.

To show this, one can invoke a model of the random function

(25) $$f\left(\theta \right)=\frac{1}{i\pi }\frac{\prod_{r=1}^{N}\left(1+i\pi f_{k,r}\left(\theta -\theta_{k,r}\right)\right)-\prod_{r=1}^{N}\left(1-i\pi f_{k,r}\left(\theta -\theta_{k,r}\right)\right)}{\prod_{r=1}^{N}\left(1+i\pi f_{k,r}\left(\theta -\theta_{k,r}\right)\right)+\prod_{r=1}^{N}\left(1-i\pi f_{k,r}\left(\theta -\theta_{k,r}\right)\right)},$$

determined by the random $\theta_{k,r}$ and $f_{k,r}$. In other words, within each period $\left(2\pi k,2\pi \left(k+1\right)\right)$ of G(t), the model function Equation (25) is characterized by 2N parameters—the positions $\theta_{k,r}$ of the roots $f(\theta_{k,r})=0$ and the derivatives $f_{k,r}=f^{\prime }\left(\theta_{k,r}\right)$ in these points, with r=1,…,N. For the phase factors entering the sum in Equation (24) this yields

(26) $$\log\frac{1-i\pi f(\theta +2\pi k)}{1+i\pi f(\theta +2\pi k)}=\sum_{r=1}^{N}\log\frac{1-i\pi f_{k,r}\left(\theta -\theta_{k,r}\right)}{1+i\pi f_{k,r}\left(\theta -\theta_{k,r}\right)}.$$

and allows one to express the probability amplitudes Equation (23) in the form

(27) $$\psi_{m}=\int_{0}^{2\pi }d\theta \frac{e^{-im\theta }}{2\pi }\prod_{r=1,k=0}^{N,n-1}\frac{1-i\pi f_{k,r}\left(\theta -\theta_{k,r}\right)}{1+i\pi f_{k,r}\left(\theta -\theta_{k,r}\right)}$$

convenient for the analysis.

Randomness of f(θ) implies averaging over an ensemble of $\theta_{k,r}$ and $f_{k,r}$ of the population

(28) $$\begin{array}{cc} \rho_{m}\left(2\pi n\right) & =\int_{0}^{2\pi }e^{\sum_{r=1,k=0}^{N,n-1}\log\frac{1-i\pi f_{k,r}\left(\theta -\theta_{k,r}\right)}{1+i\pi f_{k,r}\left(\theta -\theta_{k,r}\right)}\frac{1+i\pi f_{k,r}\left(\vartheta -\theta_{k,r}\right)}{1-i\pi f_{k,r}\left(\vartheta -\theta_{k,r}\right)}}\\ \; & e^{im\vartheta -im\theta }\frac{d\vartheta d\theta }{4\pi^{2}} \end{array}$$

of the level *m* after *n* periods of G(t) given by ${\left|\psi_{m}\right|}^{2}$ of Equation (27). One can chose an independent uniform distribution of the roots positions $\theta_{k,r}$ within the interval $\left(0,2\pi \right)$ and the uniform distribution of the derivatives $f_{k,r}$ within a band (−F,F), while the width 2F of this band serves as the parameter of the model characterizing typical front duration of the phase changes. Each term of the sum in the exponent is a statistically independent averaged factor for the population

$$\int_{-F}^{F}\frac{dy}{2F}\int_{0}^{2\pi }\frac{dx}{2\pi }\frac{1-i\pi y(\theta -x)}{1+i\pi y(\theta -x)}\frac{1+i\pi y(\vartheta -x)}{1-i\pi y(\vartheta -x)}.$$

It can be calculated separately and in the limit of large 2F where the integration limits for *x* can be extended to ±∞ and give

$$\int_{-F}^{F}\frac{dy}{2F}\left(1-\frac{4\pi (\vartheta -\theta )}{\pi y(\vartheta -\theta )+2i}\mathrm{sign}\left(y\right)\right),$$

while the second integration results in

(29) $$1-\frac{2}{F}\log\left[1+{\left(\frac{F\pi \zeta }{2}\right)}^{2}\right],$$

where ζ=ϑ−θ.

After substitution Equation (29) to Equation (28) and integration over $d\frac{\vartheta +\theta }{2}$ the last expression gives the population

$$\rho_{m}\left(2\pi n\right)=\int_{0}^{2\pi }\frac{d\zeta }{4\pi^{2}}e^{im\zeta +Nn\log\left(1-\frac{2}{F}\log\left[1+{\left(\frac{F\pi \zeta }{2}\right)}^{2}\right]\right)},$$

which after evaluation of the integral by the saddle-point method yields the diffusion profile

(30) $$\rho_{m}\left(2\pi n\right)=\frac{1}{2\pi \sqrt{2\pi NnF}}\exp\left\{-\frac{m^{2}}{8\pi^{2}F\;Nn}\right\},$$

in complete agreement with one’s intuitive expectations.

### 5.3. Irrational Frequency and Liouville Numbers

We thus identified two regimes, the linear increase of the distribution width, resulting from the constructive interference in the case of a resonance, and the diffusive regime under the action of random force where the interference is suppressed by the randomness. Is it possible to identify a regime where interference of the revivals is destructive? The answer to this question is affirmative, and the simplest example of such a case is a harmonic excitation $f\sin\left[\omega t\right]$ with a period 2π/ω incommensurate with that of G(t). This implies that not even a high order composite resonance occurs in the system under the action of perturbation. Equation (20) for this case reads

(31) $$\psi_{m}\left(2\pi r\right)=-\int_{0}^{2\pi }\frac{d\theta }{2\pi }e^{-im\theta +\sum_{k=0}^{r-1}\log\frac{1-i\pi f\sin\left[\omega (\theta +2\pi k)\right]}{1+i\pi f\sin\left[\omega (\theta +2\pi k)\right]}},$$

where ω is an irrational number. During each period of G(t), i.e., for each *k*, the phase factor may turn to zero several times at the points $\theta_{p,k}=\pi p/\omega -2\pi k$, and the derivatives in these points may take just two values ±2iπωf.

Though for long times, due to incommensurably of the periods of f(t) and G(t), the roots $\theta_{p,k}$ form a rather dense set of points in the interval (0,2π); however, this neither implies that the root density is uniform nor that the signs of derivatives are randomly distributed. The existence of correlations in the positions of the roots and in the signs of derivatives dictated by the Number Theory may and do result in the strong interference phenomena. In order to reveal this interference we cast the sum of logarithms entering Equation (31) to power series

2iIm∑k=0,m=0,n=0r−1,∞,∞(−1)n+1πf2m+n(m+n−1)!n!m!ein−mω(θ+2πk),

perform summation over r−1 periods *k* of the kernel G(t)

2iIm∑m,n=0∞(−1)n+1πf2m+n(m+n−1)!n!m!ein−mω(θ+πr−1)sin(n−m)πrωsin(n−m)πω,

make the replacements n→m+s for n>m, or m→n+s otherwise, getting

2iIm∑n,s=0∞(−1)n+1πf2s+2n(s+2n−1)!n!n+s!e−isω(θ+πr−1)sinsπrωsinsπω+2iIm∑m,s=0∞(−1)m+1πf2s+2m(s+2m−1)!n!n+s!−eiω(θ+πr−1)−ssinsπrωsinsπω,

perform summation over *n* (or *m*) noting that

$$\sum \frac{x^{m}\left(2m+s-1\right)!}{m!(m+s)!}=\frac{2^{s}}{{\left(1+\sqrt{1-4x}\right)}^{s}s},$$

and finally obtain the expression

(32) $$\begin{array}{cc} \; & 2\sum_{s=2n+1}{\left[\frac{\pi f}{1+\sqrt{1+\pi^{2}f^{2}}}\right]}^{s}\\ \; & {\left(-1\right)}^{n}\sin\left[s\omega (\theta +\pi \left(r-1\right))\right]\frac{\sin\left[s\pi r\omega \right]}{s\sin\left[s\pi \omega \right]} \end{array}$$

for the sum of logarithms in Equation (31). Here summation is performed over odd natural numbers s=2n+1. In the summand, one recognizes the combination $\frac{\sin\left[s\pi r\omega \right]}{\sin\left[s\pi \omega \right]}$ typical of the *r*-beam interference, which amounts to *r* for the resonant frequencies corresponding to zero denominators, and which is rapidly drops due to the destructive interference, when the frequency is detuned from the resonance.

The summand of Equation (32) contains three factors, the first one ${\left(\pi f/\left(1+\sqrt{1+\pi^{2}f^{2}}\right)\right)}^{s}$ exponentially decreasing with the increasing summation index and vanishing at typical sizes s∼πf, the second factor $\cos\left[s\omega (\theta +\pi \left(r-1\right))\right]$ corresponding to oscillations with a constant amplitude, and the third, interference induced factor $\frac{\sin\left[s\pi r\omega \right]}{s\sin\left[s\pi \omega \right]}$, which is the most important for our analysis. For an irrational ω, the argument of the sine in the denominator never coincides with a multiple of π, and hence the denominator never assumes zero value, such that a finite difference depending on the parameter ε defined via the relation sπω=kπ+sπε always exists. Given an integer *s*, the minimum difference $s\pi \varepsilon_{s}$ is determined by the best rational approximation $\omega =\frac{k}{s}+\varepsilon_{s}$ of the irrational frequency by a rational number with the denominator *s*.

From the explicit expression for the derivative of the phase

$$\Phi_{r}^{\prime }\left(\theta \right)=2\omega \sum_{s=2n+1}{\left[\frac{\pi f}{1+\sqrt{1+\pi^{2}f^{2}}}\right]}^{s}\sin\left[s\omega (\theta +\pi \left(r-1\right))\right]\frac{\sin\left[s\pi r\omega \right]}{\sin\left[s\pi \omega \right]}$$

by the substitution $\omega \to \frac{k}{s}+\varepsilon_{s}$ one finds

(33) $$\Phi_{r}^{\prime }\left(\theta \right)=\frac{2\omega }{{\left(-1\right)}^{r+1}}\sum_{s=2n+1}{\left[\frac{\pi f}{1+\sqrt{1+\pi^{2}f^{2}}}\right]}^{s}\sin\left[s\omega (\theta +\pi \left(r-1\right))\right]\frac{\sin\left[rs\pi \varepsilon_{s}\right]}{\sin\left[s\pi \varepsilon_{s}\right]}.$$

Equation (33) has a transparent physical meaning: each term of the sum corresponds to an *s*-photon resonance, which has an exponentially small first factor corresponding to a composite transition matrix element decreasing as $\exp\left[-s/\pi f\right]$, the second factor oscillating at the frequency of the *s*-th harmonics of the driving field, and the third factor accounting for the mismatch $s\varepsilon_{s}$ of the resonance. The exponential decrease of the composite matrix element suggests to consider at most s∼2πf first terms of the infinite sum. The third factor has a numerator less than unity in the absolute value while the denominator is not less than the minimum distance $\varepsilon_{s<2\pi f}$ between the irrational number and its best rational approximation with a denominator *s* inferior to 2πf. Apart from a zero measure set of so-called *Liouville numbers*, i.e., for a *generic* real ω, in virtue of so-called Dirichlet’s theorem, the number theory suggests an estimate for this mismatch distance $\varepsilon_{s<q}∼1/q^{2}$ while, according to Hurwitz’s theorem, the number $\sqrt{5}$ can be taken for the proportionality coefficient. One therefore can estimate the upper bound of the phase derivative Equation (33) as

(34) $$\left|\Phi_{r\to \infty }^{\prime }\right|≲\sum_{s}4\sqrt{5}\omega e^{-\frac{s}{\pi f}}\frac{s}{\pi }≃\frac{4\sqrt{5}\omega }{\pi }{\left(\pi f\right)}^{2}.$$

The stationary phase method of evaluation of the integral Equation (31) suggests to consider this quantity as an estimate of the maximum possible width *W* of the population distribution over the energy scale, and this distribution attains after $r∼1/s\varepsilon_{s<2\pi f}∼\pi f$ of the kernel periods.

One can interpret this result as a linear increase of the population distribution till the moment of time t∼r∼πf , while after this time the destructive interference of the kernel return spikes becomes important and destroys the process of the population distribution broadening. This result is known as Dynamic Localization—a universal regime for the case of monochromatic excitation, which occurs because in the general case couplings between the system and the field harmonics at the frequencies of high-order parametric resonances are no longer able to overcome the mismatches of these resonances given by the best rational approximations of the irrational frequency. Exceptions from this general case are two sets of frequencies of zero measure: a countable set of the rational frequencies considered above, for which the distribution width linearly increases in time, and a more sophisticated set that is equivalent to continuum from the point of view of the set theory, which corresponds to so-called *Liouville numbers* [7] presented now. The second set may result in time dependencies of the distribution width that are intermediate between the linearly growing and the bounded ones.

Concept of the Liouville numbers relates to the Number Theory, which attracting more and more the attention of physisists [8]. One defines these numbers via their representation

(35) $$\omega =\sum_{n}\omega_{n}b^{-n}$$

over an integer base *b* with the integer coefficients $\omega_{n}\;\in \;\left\{0,1,\ldots ,b-1\right\}$. The string of ordered coefficients $\left\{\omega_{n}\right\}$ contains infinite number of long and gradually getting longer sequences of zeros $\omega_{n\in Sj}=0$. By $S_{j}=\left\{k_{2j-1},k_{2j-1}+1,\ldots ,k_{2j}-1\right\}$ one denotes here *j*-th set of sequential integers numerating the orders *n* in the sum Equation (35) for which all the coefficients $\omega_{n}$ must vanish. Each set $S_{j}$ has a length $L_{2j-1}=k_{2j}-k_{2j-1}$ that not only monotonously increase with increasing *j* but does this not slower than the lengths $L_{2j}=k_{2j+1}-k_{2j}$ of the complimentary intervals ${\bar{S}}_{j}=\left\{k_{2j},\ldots ,k_{2j+1}-1\right\}$ where $\omega_{n\in {\bar{S}}_{j}}$ not required to be zero. A string

(36) $$\omega =1.\underset{{\bar{S}}_{1}}{\underset{︸}{101}}\underset{S_{1}}{\underset{︸}{0000}}\underset{{\bar{S}}_{2}}{\underset{︸}{11011}}\underset{S_{2}}{\underset{︸}{000000}}\underset{{\bar{S}}_{3}}{\underset{︸}{110101}}\underset{S_{3}}{\underset{︸}{00000000}}\ldots$$

where the sequences $S_{j}$ are shown explicitly gives an example of Liouville number in the binary representation b=2. Given a base *b*, each Liouville number can be defined by a set of the register positions $\left\{k_{m}\right\}$

(37) $$\omega =\omega \left(\left\{k_{m}\right\}\right)$$

where the integers $k_{2j-1}$ and $k_{2j}$ denote the beginnings of the sets $S_{j}$ and ${\bar{S}}_{j}$, respectively, while $L_{m}$ can be considered as an increasing function $L_{m}=F\left(k_{m}\right)$ of $k_{m}$. The character of F(x) increase at large arguments specifies a class of the Liouville numbers one deals with. The Liouville numbers in general have very good rational approximations—just in virtue of the assumption that F(x) monotonously increases, for a denominator $s_{j}=b^{k_{2j-1}}$, the accuracy $\varepsilon_{s_{j}}=b^{-k_{2j}}$ of the Liouville number rational approximation remains better than any power *g* approximation $\varepsilon_{s_{j}}<s_{j}^{-g}$. But when F(k) grows fast enough, the approximation may approach the exponential accuracy.

For the latter case, smallness of the denominator $\sin\left[s_{j}\pi \omega \right]≃s_{j}\pi \varepsilon_{s_{j}}$ in Equation (32) for *j*-th best approximation can compensate for the exponential smallness of the numerator $\exp\left[-s_{j}/\pi f\right]$, and than the high $s_{j}$-order parametric resonances do occur in the system since the composite transition matrix element $∼e^{-s_{j}/\pi f}$ exceeds the resonance mismatch $s_{j}\pi \varepsilon_{s_{j}}$. Moreover, the high order resonances turns to be as important as the low order resonances although they manifest themselves at much longer time scales. The leading contribution to the population distribution width

(38) $$W\left(t∼\frac{1}{2n\varepsilon_{s_{j}}}\right)∼\sum_{q=1}^{j}e^{-\frac{s_{j}}{\pi f}}\frac{1}{\sin\left[s_{j}\pi \varepsilon_{s_{j}}\right]}$$

suggested by the derivative Equation (33) where the summation goes now over the best rational approximation denominators $s_{j}$ exclusively, and the relations $s_{j}=b^{k_{2j-1}}$, $\varepsilon_{s_{j}}=b^{-k_{2j}}$, $\sin\left[s_{j}\pi \varepsilon_{s_{j}}\right]∼s_{j}\varepsilon_{s_{j}}$, yield

(39) $$W∼\sum_{q=1}^{j}b^{k_{2j}-k_{2j-1}}e^{-b^{k_{2j-1}}/\pi f}.$$

for the time moment

$$t∼\frac{1}{s_{j}\varepsilon_{s_{j}}}=b^{k_{2j}-k_{2j-1}},$$

which defines the length $L_{2j-1}=k_{2j}-k_{2j-1}$ of the longest interval $S_{j}$ and thereby the corresponding denominator $s_{j}$ of the largest *j*-th rational approximation to be taken into account.

One can alternatively parametrized the integers $k_{2j-1}$ that define a Liouville number by the time parameters

$$t_{m}=b^{k_{m}-k_{m-1}},$$

such that $L_{m}=\frac{\ln t_{m}}{\ln b}$, and $s_{j}=\prod_{m}^{2j-1}t_{m}$, which allows one to explicitly represent the augmentation $\Delta W_{2j}$ of the width Equation (39) that occurs at time $t∼\frac{1}{s_{j}\varepsilon_{s_{j}}}=t_{2j}$ by the expression

(40) $$\Delta W_{2j}=t_{2j}\exp\left[-\frac{1}{\pi f}\prod_{m=1}^{2j-1}t_{m}\right],$$

independent on the base *b*. One can see that provided each next time parameter is of the order of the exponent of the product of all former time parameters, i.e., when

$$\ln t_{n}∼\frac{1}{\pi f}\prod_{m=1}^{n}t_{m},$$

the width of the distribution may increase by an amount of the order of unity and hence it tends to infinity with the time, although, this increase is not graduate but goes in steps. Each of the steps occurs when the time $t≃t_{2j}$ becomes long enough, such that the ratio

$$\frac{\sin\left[s\pi r\omega \right]}{\sin\left[s\pi \omega \right]}=\frac{\sin\left[rs\pi \varepsilon_{s}\right]}{\sin\left[s\pi \varepsilon_{s}\right]}$$

in Equation (32) approaches an exponentially large maximum value $1/s\pi \varepsilon_{s}$ corresponding to the next best rational approximation $\varepsilon_{s}$ of the irrational frequency given by the Liouville number.

One can look at this situation from another point of view. Imagine that at a given strength of the harmonic perturbation, one has identified a multi-quantum resonance of a high order $s_{j}$, which as a consequence is very narrow having a width $\delta \omega_{2j-1}$ given by the exponentially small $∼\exp\left(-s_{j}/\pi f\right)$ composite matrix element of the multiphoton transition. Still within this narrow width $\delta \omega_{2j-1}$, one can find another resonance of even higher order, and hence even much narrower, $\delta \omega_{2j+1}\ll$ $\delta \omega_{2j-1}$. By tuning the frequency to this second resonance, one will simultaneously have two resonances that correspond to two distinct Rabi periods. The process can be continued by identifying a third resonance in the vicinity of the second one, than a forth one etc., thus gradually approaching an irrational frequency corresponding to a Liouville number and generating a string of the corresponding Rabi half-periods $\left\{t_{2j-1}\right\}$. Since the width $\delta \omega_{2j-1}$ drops exponentially with the order $s_{j}$ of the resonance, each next $s_{j}$ exponentially increases with the resonance number *j* , while the Rabi half-periods $t_{2j-1}$ increase as double exponents as the consequence. and the width $\Delta W_{2j-1}$ of the population distribution given by Equation (40).

Now one can address the question: how to choose the sequence $\left\{t_{m}\right\}$ in order to obtain any desired asymptotic dependence W(t)? This situation corresponds to a general intermediate case placed in between the limiting case of parametric resonance with a linear growth of the width and the limiting case of a generic boarded dynamically localized distribution. Assuming a power-law dependence $\Delta W=t^{\alpha }$ with α<1, from Equation (40) one finds the recurrent relation

$$\ln t_{n}=\frac{1}{\left(1-\alpha \right)\pi f}\prod_{m=1}^{n-1}t_{m}$$

for the time parameters solving this problem. Due to the rapid growing of $\Delta W_{2j-1}$ with the index *j*, the width $W_{2j-1}$ of the population distribution is mainly given by the last increment $\Delta W_{2j-1}$, and hence the recurrent relation

$$\ln\frac{t_{n}}{W(t_{n})}=\frac{1}{\pi f}\prod_{m=1}^{n-1}t_{m}$$

solves the problem for the general case. The fact that the left hand side of the last equation must be positive, implies that in the asymptotic regime *W* grows with time slower than linearly.

### 5.4. Several Incommensurable Irrational Frequencies

In the case of excitation by a multi-harmonic external field f(t)=f(sinωt+sinνt+… +sinμt) with a number *M* of irrational incommensurable frequencies ω, ν… and μ, Equation (23) written for the probability amplitudes at the time moment corresponding to *k*-th period has the phase term

$$\Phi \left(\theta ,r\right)=-i\sum_{k=0}^{r-1}\log\frac{1-i\pi f\left[\sin\omega (\theta +2\pi k)+\ldots +\sin\mu (\theta +2\pi k)\right]}{1+i\pi f\left[\sin\omega (\theta +2\pi k)+\ldots +\sin\mu (\theta +2\pi k)\right]},$$

which now governs the excitation dynamics to be analyzed in the asymptotic of large *r*. We cast each of *r* terms of the sum to multiple Fourier series by noting that

$$-i\log\frac{1-i\pi f\left[\sin x+\sin y+\ldots +\sin z\right]}{1+i\pi f\left[\sin x+\sin y\ldots +\sin z\right]}=\sum_{n,l\ldots ,m}c_{n,l\ldots ,m}e^{inx+ily+\ldots +imz},$$

where the coefficients

(41) $$c_{n,l\ldots ,m}=\int \frac{dx}{{\left(2\pi \right)}^{1-M}}\frac{e^{-\left|x\right|}}{2x}J_{n}\left(\pi fx\right)J_{l}\left(\pi fx\right)\ldots J_{m}\left(\pi fx\right)$$

are be found with the help of the Fourier representation

$$-i\log\frac{1-iX}{1+iX}=2\arctan X=\frac{1}{2\pi }\int \frac{ie^{-\left|x\right|}}{2x}e^{iXx}dx$$

and the definition of the Bessel function

$$2\pi J_{n}\left(Y\right)=\int_{0}^{2\pi }e^{iY\sin y-iny}dy,$$

we then change the order of summations

$$\Phi \left(\theta ,r\right)=\sum_{n,l\ldots ,m}c_{n,l\ldots ,m}\sum_{k=0}^{r-1}e^{i\left(n\omega +l\nu +\ldots +m\mu \right)\left(\theta +2\pi k\right)},$$

and arrive at

(42) $$\Phi \left(\theta ,r\right)=\sum_{n,l\ldots ,m}c_{n,l\ldots ,m}e^{i(\theta +\pi (r-1))(n\omega +l\nu +\ldots +m\mu )}\frac{\sin\left[\pi r(l\nu +n\omega +\ldots +m\mu )\right]}{\sin\left[\pi (l\nu +n\omega +\ldots +m\mu )\right]}.$$

The Fourier coefficients $c_{n,l\ldots ,m}$ for the case M≫1 can estimated as follows. The Bessel functions in Equation (41) reach their maximum absolute values for the arguments of the order of the indices. For smaller arguments they rapidly drop to zero, and for larger arguments they oscillate with the amplitude decreasing like a square root of the argument. Therefore, the main contribution to the integral Equation (41) comes from the domain

$$x∼\frac{\max\left(n,l,\ldots ,m\right)}{\pi f},$$

of a length of the order of the oscillation period 1/πf, where the integrand is of the order of

$$\frac{e^{-\left|x\right|}}{2x}\frac{{\left(2\pi \right)}^{M}}{{\left(\pi fx\right)}^{\left(M-1\right)/2}},$$

thus resulting in

$$c_{n,l\ldots ,m}∼\frac{e^{-\max\left(n,l,\ldots ,m\right)/\left|\pi f\right|}}{4\pi }{\left(\frac{2\pi }{\sqrt{\max\left(n,l,\ldots ,m\right)}}\right)}^{M+1},$$

Now analyzing the sum (42), one sees that on one hand side, the Fourier coefficients $c_{n,l\ldots ,m}$ are exponentially decreasing with the size of the maximum index, but on the other hand side, the interference factors

$$\frac{\sin\left[\pi r(l\nu +n\omega +\ldots +m\mu )\right]}{\sin\left[\pi (l\nu +n\omega +\ldots +m\mu )\right]}$$

may become rather large when these indices tend to infinity, such that the best rational approximation of irrational numbers may become very small. In contrast to (32), the approximation concerns not just one but a sum of *M* rational numbers multiplied by integer coefficients. Indeed, given l,n,… and *m*, the fractional part $\varepsilon_{n,l\ldots ,m}$ of the irrational number

$$l\nu +n\omega +\ldots +m\mu =k+\varepsilon_{n,l\ldots ,m}$$

determines the maximum size of the interference factor

(43) $$\left|\frac{\sin\left[\pi r(l\nu +n\omega +\ldots +m\mu )\right]}{\sin\left[\pi (l\nu +n\omega +\ldots +m\mu )\right]}\right|≲\frac{1}{\pi \left|\varepsilon_{n,l\ldots ,m}\right|},$$

which takes the maximum value at $r≃{\left|\varepsilon_{n,l\ldots ,m}\right|}^{-1}/2$.

Now one has to analyze the behavior of the inverse of the fractional part $\varepsilon_{n,l\ldots ,m}$ of an irrational number lν+nω+…+mμ as a function of the maximum value of the indices. This quantity is a very irregular function of the indices l,n,… and *m*: it takes very large values for some specific points $\left\{l_{s},n_{s},\ldots m_{s}\right\}$ that sparsely distributed over the *M*-dimensional manifold of integers, while for other points on this “hyperplane” lν+nω+…+mμ the inverse of $\varepsilon_{n,l\ldots ,m}$ remains of the order of unity . Therefore, the summation in (42) can be taken only over these specific indices, because only their contribution may result in the energy distribution of the width

(44) $$\sqrt{\bar{\left|m^{2}\right|}}∼\Phi_{\theta }^{\prime }\left(\theta ,r\right)∼\sum_{n,l\in \cup \left\{l_{s},n_{s},\ldots m_{s}\right\}}c_{n,l\ldots ,m}e^{i(\theta +\pi (r-1))(n\omega +l\nu +\ldots +m\mu )}\left(l\nu +n\omega +\ldots +m\mu \right)\frac{\sin\left[\pi r(l\nu +n\omega +\ldots +m\mu )\right]}{\sin\left[\pi (l\nu +n\omega +\ldots +m\mu )\right]}$$

growing in the course of time t∼r. Again, as it was in the case of the excitation at a single frequency given by a Liouville number, this broadening augments not gradually but by steps, whereas each next step *s* manifests itself at a time moment $t∼r≃{\left|\varepsilon_{l_{s},n_{s},\ldots m_{s}}\right|}^{-1}/2$ resulting from the contribution of a specific point $\left\{l_{s},n_{s},\ldots m_{s}\right\}$ that corresponds to the best integer approximation of lν+nω+…+mμ, yet improves with the rising *s* and thus resulting in decrease of the denominator $\sin\left[\pi (l\nu +n\omega +\ldots +m\mu )\right]$. These very points bring the factor ${\left(\pi \varepsilon_{n,l\ldots ,m}\right)}^{-1}$ large enough to compensate for the exponentially small $c_{n_{s},l_{s}\ldots ,m_{s}}$. In other words, each specific point $\left\{l_{s},n_{s},\ldots m_{s}\right\}$ of the hyperplane gives contribution at a specific time scale $r≃{\left|\varepsilon_{l_{s},n_{s},\ldots m_{s}}\right|}^{-1}/2$, while the delay among the sequential broadening events increases exponentially in time.

Now the question is whether in course of time the width of the population distribution increases unlimitedly, as it was in the case of a random f(t), or it remains localized, as in the case of a generic harmonic excitation? In order to answer this question one could formulate the best rational approximation problem in physical terms by figuring out what is the smallest detuning of the combinational frequency lν+nω+…+mμ for a maximum order $K≲M\max\left(n,l,\ldots ,m\right)$ of the resonance. The discrepancy $\varepsilon_{K}=\min\varepsilon_{n,l\ldots ,m<K/M}$ of the best integer approximation of lν+nω+…+mμ given l,n,…,m<K/M corresponds to the minimum detuning of the resonance of the order inferior to *K*. An unlimited increasing width of the population distribution given by the condition

(45) $$\sum_{n,l\ldots m\in \cup \left\{l_{s},n_{s},\ldots m_{s}\right\}}c_{n_{s},l_{s}\ldots ,m_{s}}\frac{l_{s}\nu +n_{s}\omega +\ldots +m_{s}\mu }{\pi \varepsilon_{l_{s},n_{s},\ldots m_{s}}}\to \infty$$

implies $\varepsilon_{K}$ decreasing with *K* faster than $c_{n,l\ldots ,m<K/K}∼e^{-K/M\left|\pi f\right|}{\left(K/M\right)}^{-M/2}$. This should never be the case for generic real frequencies ν,ω…,μ, since the Number Theory states that $\varepsilon_{K}=\min\varepsilon_{n,l\ldots ,m<K/M}$ scales as a power ${\left(K/M\right)}^{-M}$ of the maximum integer size $\max\left(n,l,\ldots ,m\right)∼K/M$, and hence

(46) $$\begin{array}{cc} \sqrt{\bar{\left|m^{2}\right|}} & ∼\sum_{K}\frac{M}{\pi }e^{-\frac{K}{M\left|\pi f\right|}}{\left(\frac{K}{M}\right)}^{\frac{M-1}{2}}\\ \; & ∼{\left(\pi f\right)}^{\frac{M+1}{2}}\frac{M^{2}}{\pi }\Gamma \left(\frac{M+3}{2}\right)<\infty , \end{array}$$

where $\Gamma \left(x\right)$ is the Euler gamma-function. This expression means that though the asymptotic distribution width $\sqrt{\bar{\left|m^{2}\right|}}$ remains finite, it scales exponentially with the number *M* of distinct frequencies in the Fourier decomposition of the driving field.

## 6. Conclusions

In the framework of an exactly soluble model, we identified four distinct asymptotic cases of the dynamics under the action of a time dependent perturbation: (i) the resonant case, including the parametric resonance, which is specific of the equidistant spectrum yielding the population distribution linearly broadening in time, (ii) diffusive broadening, typical of the excitation by a random perturbation, and two regimes of localization, (iii) the strong localization, where the population distribution remains of a finite width, despite the harmonic excitation, and (iv) the weak localization, where the width of the distribution grow very slowly, at most logarithmically, although some other regimes may be realized by a proper choice of frequencies. The regime (i) is specific for the chosen model of the equidistant levels, while three other regimes are likely universal since they correspond to the length of the Magnus series exceeding the number of the populated levels, where all higher order commutators are presented with equal importance.

## Data Availability

Not applicable.
